# Angiotensinogen Gene Variation and Hypertension in a Cohort Study in Japanese

**DOI:** 10.2188/jea.11.115

**Published:** 2007-11-30

**Authors:** Takuji Kishimoto, Akihiko Suyama, Atsusi Igarashi, Yoneatsu Osaki, Mikizoh Okamoto, Toshiyuki Yamamoto, Eiji Nanba, Yoichi Kurosawa, Soji Fukumoto

**Affiliations:** 1 Department of Hygiene, Faculty of Medicine, Tottori University.; 2 Gene Research Center, Tottori University.; 3 Department of Public Health, Faculty of Medicine, Tottori University.; 4 Department of Parasitology, Faculty of Medicine, Tottori University.

**Keywords:** angiotensinogen, polymorphism, hypertension, genotype, cohort study

## Abstract

Many recent case-control studies have suggested a significant relationship between M235T (the substitution of threonine for methionine at position 235 codon) polymorphism of the angiotensinogen (AGT) gene and hypertension. To investigate whether the M235T polymorphism of AGT gene affects the incidence of hypertension, a retrospective cohort study was performed among Japanese workers. The subjects were Japanese workers at an occupational site in Shimane Prefecture in Japan. The baseline data were set at the received regular health examination in 1992, and a retrospective cohort study was performed for analyzing the incidence of hypertension in 1998. The rates of M235M (MM), M235T (MT) and T235T (TT) genotypes were 4%, 32% and 64%, respectively. The relative risks of MT and TT against MM for the incidence of hypertension by single variance analysis were 1.47 [95% confidence interval (CI) 0.50 - 4.33] and 1.35 (95% CI 0.47 - 3.90), respectively. The relative risks of MT and TT against MM for the incidence of hypertension, adjusted for sex, age, body mass index, fasting glucose and cigarette smoking, drinking and exercise in 1992, were 1.49 (95% CI 0.49 - 4.53) and 1.25 (95% CI 0.42 - 3.74), respectively. The data from this study suggest that the M235T polymorphism of AGT gene has a weak role in the manifestation of hypertension. Further comprehensive studies are needed to resolve this issue.

## INTRODUCTION

Essential hypertension is a multiple factorial disease that is complicated by the interaction of genetic factors and environmental factors^1^^)^. The angiotensinogen (AGT) gene variant, M235T (mutation to the threonine of the methionine in amino acid codon 235), was reported in the pathogenesis of essential hypertension by Jeunemaitre et al.^2^^)^ for the first time in 1992. However there have been subsequently many studies investigating the hypothesis that this M235T is one of the genetic factors of essential hypertension; the hypothesis remains controversial^3^^-^^7^^)^.

Most of the reports regarding the relationship between M235T and hypertension used the method of a case - control study method that included a relatively small sample size. If the study design is not carefully conducted, a case - control study has many kinds of bias. It is very difficult to choose a suitable control - group. Thus, we carried out a retrospective cohort study with a large sample size to clarify the involvement of M235T in hypertension.

## SUBJECTS AND METHODS

*Subjects;* The research was approved by the ethics committee of the Faculty of Medicine, Tottori University and informed consent was obtained from 2042 workers in Shimane Prefecture in Japan who received their regular health examination in 1998. Excluding people who had hypertension in 1992, 1001 workers who had received the regular health examination in both 1992 and 1998 were included in this study. A retrospective cohort study for the incidence of hypertension was conducted among 1001 workers from 1992 to 1998. The following selection criteria for hypertension were applied: (a) hypertension as defined by a systolic blood pressure (SBP) ≧ 140 mmHg and / or a diastolic blood pressure (DBP) ≧ 90 mmHg; (b) absence of secondary hypertension; (c) absence of renal disease and renal insufficiency.

*Various measurements and survey of life habits;* Body mass index (BMI) was calculated by the following formula: (weight kg)/(height m)^2^. The blood pressure was measured in the right upper arm with a standard sphygmomanometer in a sitting position. The 1st sound and the 5th sound of the Korotkoff sound were used as SBP and DBP, respectively. The measurement of the fasting blood sugar value was carried out using an enzymatic method (Hexokinase, G-6-PDH, Wako, Tokyo). Smoking history (never-, ex- or active smoker), drinking habits (never-, sometime- or everyday drinker) and exercise (never taking-, sometimes taking- or always taking exercise) were assessed using a questionnaire.

*Identification of polymorphism;* Genome DNA was prepared from white blood cells with the use of a fully automatic nucleic acid extractor (MFX - 2000, TOYOBO CO., LTD., Osaka). Mutant allele specific amplification (MASA) method was used for the analysis of the polymorphism^8^^)^. Four primers were designed. The normal sense primer and antisense primer were 5′-AAGACTGGCTGCTCCCTGAT-3′ and 5′-GCTGTCCACACTGGCTCCCG-3′, respectively. The wild type primer and polymorphism primer including the mutation part were 5′-AAGACTGGCTGCTCCCTGAT-3′ and 5′-AAGACTGGCTGCTCCCTGAC-3′, respectively. Polymerase chain reaction (PCR) was performed in TaKaRa PCR Thermal Cycler (Takara Co., Tokyo) in a 10µ l reaction volume containing 10 mMol / Liter Tris-HCl (pH 8.3), 50 mMol / Liter KCl, 25µ Mol / Liter of each dNTPs, 1.25µ Mol / Liter sense primer, 1µ Mol / Liter antisense primer, 2µ Mol / Liter wild type primer, 2µ Mol / Liter polymorphism primer, 0.1U Ampli-Taq DNA polymerase (Perkin Elmer, New Jersey, USA) and 1.5 mMol / Liter MgCl_2_. The initial denaturation for 3 min at 95°C was followed by 35 cycles of denaturation for 30 sec at 95°C, annealing for 30 sec at 65°C, and extension for 60 sec at 72°C. PCR products were electrophoresed in 6% polyacrylamide gels on the Model Cassette Electrophoresis Unit “DPC” (Daiichi Pure Chemical Co., LTD, Tokyo) and DNA was visualized by ethidium bromide staining.

*Statistical analysis;* SPSS software (version 8.0, SPSS Japan Inc., Tokyo) was used for all statistical comparisons. The *χ*^2^ statistic was calculated to test the distribution trend of each index by genotype. Relative risks were calculated by logistic regression analysis method.

## RESULTS

The characteristics of subjects by sex in 1992 were shown in Table 1. There were statistically significant differences in all mean values between male and female. The proportions of the genotype found in this retrospective cohort study for MM, MT and TT were 4%, 32% and 64%, respectively (Table 2). The allele frequency of 235T was 0.801. There was no significant difference between the normotensive and hypertensive subjects in terms of the proportions of the genotypes in 1998. As shown in Table 3, MT showed a slightly lower trend of mean age, but it was not significant. No significant differences were found among the other indices. Table 4 shows the distribution of BMI, SBP, DBP and fasting glucose related to genotypes in 1998. The rates of BMI value of 26.0 or more in MM, MT and TT were 16.3% 18.9% and 16.0%, respectively. There was no distribution difference in terms of genotypes in regard to the BMI value, SBP, DBP or fasting glucose.

**Table 1.** Characteristics of subjects by sex at baseline.

Characteristics	male			female			total		
		
	No.	mean	SD	No.	mean	SD	No.	mean	SD
Age (y)	578	36.1 *	9.3	423	38.0	18.0	1001	36.9	9.5
Body mass index (Kg/m^2^)	578	22.5 *	2.8	422	21.7	15.6	1000	22.2	2.8
Systolic blood pressure (mm Hg)	578	114.4 *	10.5	422	110.1	84.0	1000	112.6	10.9
Diastolic blood pressure (mm Hg)	578	73.8 *	8.0	421	70.0	50.0	999	72.2	8.3
Fasting glucose (mg/dl)	568	95.7 *	15.7	415	90.6	63.0	983	93.5	13.3

* P < 0.002, male versus female.

**Table 2.** Number and distribution of subjects related to angiotensinogen codon 235 genotypes.

	genotype			total

	MM	MT	TT	
hypertension *	4	49	90	143
	( 3%	34%	63% )	
normotension	33	275	550	858
	( 4%	32%	64% )	

total	37	324	640	1001
	( 4%	32%	64% )	

* a systolic blood pressure ≧ 40 mmHg and / or a diastolic blood pressure ≧ 90 mmHg, and anti-hypertensive treatment

**Table 3.** Characteristics of subjects for angiotensinogen codon 235 genotypes at baseline.

Characteristics	No. of subjects MM/MT/TT	Angiotensinogen genotype						p value

		MM		MT		TT		
		mean	SD	mean	SD	mean	SD	
Age (y)	37 (4%)/324 (32%)/640 (64%)	37.6	10.1	35.9	9.4	37.4	9.4	0.06
Body mass index (Kg/m^2^)	37/324/639	22.4	2.7	22.0	2.9	22.2	2.7	0.48
Systolic blood pressure (mm Hg)	37/324/639	109.5	8.3	112.6	10.6	112.7	11.2	0.22
Diastolic blood pressure (mm Hg)	37/324/638	69.8	7.5	72.2	8.6	72.3	8.2	0.19
Fasting glucose (mg/dl)	37/319/627	93.7	11.5	94.7	16.3	92.9	11.7	0.15

**Table 4.** Distribution of various indices related to angiotensinogen codon 235 genotypes in 1998.

Characteristics	No. of subjects MM/MT/TT	Angiotensinogen genotype						Total		p value

		MM		MT		TT				
			
		No.	%	No.	%	No.	%	No.	%	
Body mass index	<18 kg/m^2^	1	2.7	13	4.0	15	2.3	29	2.9	0.5377
	18-<20 kg/m^2^	7	18.9	44	13.6	74	11.6	125	12.5	
	20-<22 kg/m^2^	7	18.9	82	25.3	136	21.3	225	22.5	
	22-<24 kg/m^2^	8	21.6	74	22.8	172	26.9	254	25.4	
	24-<26 kg/m^2^	7	18.9	59	18.2	139	21.7	205	20.5	
	≥26 kg/m^2^	7	18.9	52	16.0	104	16.3	163	16.3	
	total	37	100.0	324	100.0	640	100.0	1001	100.0	

Systolic blood pressure	<140 mmHg	35	94.6	296	91.4	586	91.6	917	91.6	0.6517
	140 - <160 mmHg	2	5.4	24	7.4	51	8.0	77	7.7	
	≥160 mmHg			4	1.2	3	0.5	7	0.7	
	total	37	100.0	324	100.0	640	100.0	1001	100.0	

Diastolic blood pressure	<90 mmHg	35	94.6	288	88.9	578	90.3	901	90.0	0.3462
	90 - <95 mmHg	1	2.7	23	7.1	49	7.7	73	7.3	
	≥95 mmHg	1	2.7	13	4.0	13	2.0	27	2.7	
	total	37	100.0	324	100.0	640	100.0	1001	100.0	

Fasting glucose	<126 mg/dl	36	97.3	310	95.7	623	97.3	969	96.8	0.5658
	126 - < 140 mg/dl	1	2.7	7	2.2	10	1.6	18	1.8	
	≥140 mg/dl			7	2.2	7	1.1	14	1.4	
	total	37	100.0	324	100.0	640	100.0	1001	100.0	

In regard to the relative risk (RR) for hypertension, the RRs of MT and TT against MM by univariate analysis were 1.47 and 1.35, respectively (Table 5, Model 1). These RRs showed a slightly higher trend, but it was not significant. In both cases that sex and age were adjusted (Table 5, Model 2) and in sex, age and BMI adjusted analysis (Table 5, Model 3), RRs of MT against MM were slightly higher in comparison with Model 1, but these were not significant. When the multiple risk factors of sex, age, BMI, fasting glucose, cigarette smoking, drinking habit and exercise were adjusted, RRs of MT and TT against MM were 1.49, 1.25 respectively. Although these were not significant, a weak positive relationship was found between polymorphism and hypertension.

**Table 5.** Hypertension risk estimates associated with angiotensionogen M235T gene polymorphism and established risk factors based on logistic regression analysis.

Risk variables	Relative Risk (95% CI)		p value
Angiotensinogen polymorphism			
Model 1 (univariate)			
MT vs MM	1.47	(0.50, 4.33)	0.49
TT vs MM	1.35	(0.47, 3.90)	0.58
Model 2 (adjusted for sex, age)			
MT vs MM	1.53	(0.50, 4.67)	0.45
TT vs MM	1.28	(0.43, 3.82)	0.66
Model 3 (adjusted for sex, age, BMI)			
MT vs MM	1.53	(0.50, 4.67)	0.45
TT vs MM	1.29	(0.43, 3.85)	0.65
Model 4 (adjusted for multiple risk factors)			
MT vs MM	1.49	(0.49, 4.53)	0.48
TT vs MM	1.25	(0.42, 3.74)	0.68

Multiple risk factors introduced into model 4			
Sex Male vs female	1.65	(1.14, 2.41)	0.009
Age	1.06	(1.04, 1.08)	<0.0001
BMI	1.15	(1.08, 1.22)	<0.0001
Fasting glucose	1.02	(1.01, 1.03)	0.0025
Cigarette smoking			
Ex-smokers vs Never-smoked	1.21	(0.55, 2.69)	0.64
Active-smokers vs Never-smoked	0.81	(0.55, 1.18)	0.27
Drinking			
Sometime vs Never	0.60	(0.39, 0.92)	0.64
Everyday vs Never	0.73	(0.47, 1.14)	0.27
Exercise			
Sometime vs Always	0.88	(0.43, 1.84)	0.74
Never vs Always	1.02	(0.54, 1.91)	0.94

## DISCUSSION

In the population examined in this study, the frequency of the wild type MM was the lowest, while that of the mutant type TT was the highest. There were no significant differences in the BMI, SBP, DBP and fasting glucose between the genotypes. The RRs of MT and TT against MM for hypertension analyzed by correction using various factors were not significant, but there were weak correlations (1.49 and 1.25, respectively).

The allele frequency in the population was higher than that of Caucasians^2^^)^. This agreed with the results reported in many studies on investigation of the allele frequency in Japanese subjects^9^^-^^13^^)^. The allele frequency was 0.801 in the present study, and ranged from 0.604 to 0.860 in published studies. This wide range of the allele frequency is probably because there are regional differences in the genetic background in Japan.

The relationship between this genetic polymorphism and hypertension has been suggested in studies performed in Japan and other countries^1^^-^^7^^)^. Kato et al. were the first to cast doubt on the relationship^14^^)^. The different results for the relationship between genotypes and hypertension may be caused by the possibility of bias in choosing controls, differences between races, regional differences in the genetic background, differences in the age of subjects and multifactors of the examined disease, because most of the studies were hospital- based case-control studies in a small sample size^15^^)^. Recently, Kato et al. performed a case-control study in a large sample size using the four renin-angiotensin gene including M235T, and they concluded that there was no significant evidence for disease association^16^^)^.

To improve on the drawbacks of case-control studies in a small sample size, we performed a cohort study in a large sample size and obtained weak correlations between genotypes and hypertension. This study was a retrospective cohort study, in which bias could be corrected to some extent, but the adequacy of the results may be low in this study compared to a prospective cohort study. Analyses were performed using 4 models, and higher RRs of MT against MM were shown by the multivariate analyses than by the univariate analysis. It was considered to be necessary to evaluate which factors should be corrected. It was also considered necessary to investigate publication bias^14^^)^.

In conclusion, there was a weak correlation between the M235T polymorphism of the AGT gene and hypertension in a retrospective cohort study corrected for various factors in Japanese subjects performed between 1992 and 1998. We consider it necessary to perform further investigations while evaluating the factors to be corrected and the possibility of prospective cohort studies.
